# 

**Published:** 1855-02

**Authors:** 


					﻿No. IX.]
FEBRUARY.
[Vol. x" '
BUFFALO
MEDICAL JOURNAL
AND
*
Jltontfoh) llcuiciv
OF
MEDICAL AND SURGICAL SCIENCE.
AUSTIN FLINT, M. !>..	>
SANFORD li. HUNT. M. D„ ) Ed,tors»
BUFFALO, N. Y.:
PUBLISHED BY THOMAS 4 LATUROP&
Commercial Advertiwr Buil>liDk’».
1855.
Price 82.50 per annum, in advance.
				

